# Optimizing avian flight dynamics with a synergetic bio-inspired and machine learning approach

**DOI:** 10.3389/frobt.2026.1788627

**Published:** 2026-04-07

**Authors:** Waleed Khalid, Valentina Leontiuk, Innokentiy Kastalskiy, Victor B. Kazantsev

**Affiliations:** 1 Laboratory of Neurobiomorphic Technology, Moscow Institute of Physics and Technology (MIPT), Dolgoprudny, Moscow, Russia; 2 Department of Neurotechnology, Lobachevsky State University of Nizhny Novgorod (UNN), Nizhny Novgorod, Russia; 3 Russian State Scientific Center for Robotics and Technical Cybernetics (RTC), St Petersburg, Russia

**Keywords:** bio-inspired optimization, biomimetics, bird flight, computational fluid dynamics (CFD), computational modeling, hybrid modeling, machine learning, numerical simulation

## Abstract

This study presents a composite numerical and machine learning framework to enhance the aerodynamic performance of a bio-inspired flapping wing. The wing kinematics were extracted from biological flight data using a multi-step process: the DeepLabCut tool was applied to extract body point coordinates from avian flight videos, followed by data digitization in Google Colab and trajectory post-processing in Python. These kinematics were then prescribed to a three-dimensional wing model for high-fidelity unsteady Reynolds-Averaged Navier-Stokes (URANS) simulations of incompressible turbulent flow in ANSYS Fluent, utilizing a user-defined function (UDF) and a sliding mesh technique. Validated against existing experimental data, the numerical model serves as a reliable data generation tool. Subsequently, a machine learning model was developed to explore the design space and identify kinematic parameters that optimize aerodynamic efficiency. The results demonstrate that the proposed framework effectively bridges biological observation with computational optimization, offering a robust approach for the performance enhancement of bio-inspired flapping wings.

## 1 Introduction

Biomorphic robotic drones have become indispensable across numerous fields due to their lightweight construction, energy-efficient operation in confined spaces, agile maneuverability, and inherent collision resilience (De Croon, 2020; Lane et al., 2020; Manabendra et al., 2024). The growing interest in biomimetic engineering reflects the pursuit of next-generation unmanned aerial systems with superior performance characteristics. Within this domain, flapping-wing mechanisms that emulate avian or insect flight have attracted particular attention, with dynamic wing deformation, especially twisting, identified as a key determinant of aerodynamic efficiency.

Research in flapping-wing aerodynamics consistently highlights dynamic twist as a critical factor in enhancing aerodynamic performance. Studies confirm the advantages of biomimetic kinematic patterns, such as synchronized flapping and folding, in improving propulsive efficiency (Yan et al., 2025). The potential of controlled deformation has been further quantified in studies demonstrating that prescribed three-dimensional wing motion can yield substantial increases in thrust and efficiency relative to rigid wings (Tay, 2016). Experimental investigations on micro air vehicles provide supporting evidence: flexible twisting wings have been shown to increase aerodynamic efficiency by 41.3% and lift by 35.3%, revealing the limitations of quasi-steady modeling assumptions (Colmenares et al., 2020).

Numerical simulations have played an instrumental role in elucidating the underlying flow mechanisms. Recent work attributes the performance gains associated with wing twist to modifications in vortex dynamics, including the stabilization of the leading-edge vortex (Bouard et al., 2024). However, the optimization of kinematic parameters remains a complex task. While asymmetric flapping might seem intuitively advantageous, a numerical study revealed that time-averaged performance is predominantly governed by the mean angle of attack, suggesting that asymmetric hovering does not inherently improve efficiency (Diaz-Arriba et al., 2021). Moreover, the manner in which deformation is implemented proves critical: comparative studies of different deformation modes conclude that coordinated bending–twisting coupling yields greater performance improvements than either mode in isolation (Kumar et al., 2024). Collectively, these findings underscore the importance of dynamic wing morphing as a mechanism for augmenting lift and reducing drag (Kaygan and Ulusoy, 2018; Kelayeh and Djavareshkian, 2021; Moore, 1986; Camacho et al., 2024).

Despite these advances, challenges related to scalability and real-world deployment persist. A key limitation in accurately simulating and optimizing such systems is the reliance of conventional computational fluid dynamics (CFD) approaches on static or predefined geometries, which restricts their capacity to capture unsteady, dynamically coupled phenomena. Aerodynamic optimization under realistic operating conditions thus remains a complex and unresolved challenge, particularly for wings subjected to turbulent flows.

A recent study by Khalid et al. (2025) investigates the aerodynamic consequences of a half-twisting mechanism in a flapping wing. The results indicate that introducing controlled twist during the upstroke effectively attenuates the instantaneous lift coefficient; importantly, however, the cycle-integrated lift remains positive, contributing to an overall enhancement of aerodynamic performance. Furthermore, numerical simulations exhibit strong agreement with an accompanying mathematical model, confirming the consistency of the proposed framework.

In the present study, we address the aforementioned limitation by integrating time-varying wing geometry with a User-Defined Function (UDF) to prescribe dynamic motion within the commercial CFD solver ANSYS Fluent. This approach enables a more realistic representation of wing kinematics and facilitates the computation of transient aerodynamic forces under dynamically evolving conditions.

The objective of this work is to investigate the aerodynamic effects of a flapping wing executing simultaneous twisting motion. The wing oscillates asymmetrically between +30° and −20° while undergoing a twist of very small amplitude; the kinematic parameters are derived from real bird flight data extracted via machine learning techniques. Our goal is to maximize the positive integral of lift and thrust coefficients over the flapping cycle. Achieving this would demonstrate how such kinematic regimes can sustain efficient flight, replicating the aerodynamic advantages observed in avian systems.

## 2 Mathematical modeling

Three-dimensional, incompressible, dynamic turbulent simulations are performed (Williams, 1969). The governing equations for mass and momentum conservation in component-wise form are:

$$\nabla \cdot u=0\;\text{or}\;\frac{\partial u_{i}}{\partial x_{i}}=0$$ (1)

$$\frac{\partial u_{i}}{\partial t}+u_{j}\frac{\partial u_{i}}{\partial x_{j}}=-\frac{1}{\rho }\frac{\partial p}{\partial x_{i}}+\nu \frac{\partial^{2}u_{i}}{\partial x_{j}\partial x_{j}}+g_{i}$$ (2)

where $u_{i}$ (with i=1,2,3 ) represents the velocity components in the x , y , and z directions, respectively and $g_{i}$ is the i -th component of the gravitational acceleration vector g as shown in Equations 1, 2.

Let any flow variable ϕ be decomposed into mean and fluctuating parts (Girimaji, 2006) as shown in the Equations 3, 4.

$$\phi \left(x,t\right)=\bar{\phi }\left(x\right)+\phi^{\prime }\left(x,t\right)$$ (3)

where the Reynolds (time) average is defined as:

$$\bar{\phi }\left(x\right)=\underset{T\to \infty }{\lim}\frac{1}{T}\int_{t_{0}}^{t_{0}+T}\phi \left(x,t\right)dt$$ (4)

Substituting $u_{i}={\bar{u}}_{i}+u_{i}^{\prime }$ and $p=\bar{p}+p^{\prime }$ into the continuity equation yields:

$$\frac{\partial \left({\bar{u}}_{i}+u_{i}^{\prime }\right)}{\partial x_{i}}=0$$ (5)

Taking the Reynolds average of this equation:

$$\bar{\frac{\partial \left({\bar{u}}_{i}+u_{i}^{\prime }\right)}{\partial x_{i}}}=0$$ (6)

and applying the averaging rules gives the mean continuity equation:

$$\frac{\partial {\bar{u}}_{i}}{\partial x_{i}}=0\;\left(\text{Mean continuity}\right)$$ (7)

Similarly, substituting (Equations 5, 6) in Equation 2 the decomposed variables into the momentum equation:

$$\frac{\partial \left({\bar{u}}_{i}+u_{i}^{\prime }\right)}{\partial t}+\left({\bar{u}}_{j}+u_{j}^{\prime }\right)\frac{\partial \left({\bar{u}}_{i}+u_{i}^{\prime }\right)}{\partial x_{j}}=-\frac{1}{\rho }\frac{\partial \left(\bar{p}+p^{\prime }\right)}{\partial x_{i}}+\nu \frac{\partial^{2}\left({\bar{u}}_{i}+u_{i}^{\prime }\right)}{\partial x_{j}\partial x_{j}}+g_{i}$$ (8)

Expanding the convective term:

$$\left({\bar{u}}_{j}+u_{j}^{\prime }\right)\frac{\partial \left({\bar{u}}_{i}+u_{i}^{\prime }\right)}{\partial x_{j}}={\bar{u}}_{j}\frac{\partial {\bar{u}}_{i}}{\partial x_{j}}+{\bar{u}}_{j}\frac{\partial u_{i}^{\prime }}{\partial x_{j}}+u_{j}^{\prime }\frac{\partial {\bar{u}}_{i}}{\partial x_{j}}+u_{j}^{\prime }\frac{\partial u_{i}^{\prime }}{\partial x_{j}}$$ (9)

The last term can be rewritten using the product rule:

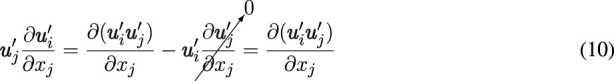

where the cancellation follows from the continuity equation for fluctuations.

Applying Reynolds averaging rules yields the final form Equations 8–10 of the Reynolds-Averaged Navier-Stokes (RANS) equations:

$$\frac{\partial {\bar{u}}_{i}}{\partial t}+{\bar{u}}_{j}\frac{\partial {\bar{u}}_{i}}{\partial x_{j}}=-\frac{1}{\rho }\frac{\partial \bar{p}}{\partial x_{i}}+\nu \frac{\partial^{2}{\bar{u}}_{i}}{\partial x_{j}\partial x_{j}}-\frac{1}{\rho }\frac{\partial \bar{u_{i}^{\prime }u_{j}^{\prime }}}{\partial x_{j}}+g_{i}$$ (11)

The Reynolds stress tensor is symmetric as shown in Equation 11 and defined as:

$$\bar{u_{i}^{\prime }u_{j}^{\prime }}=\left[\begin{array}{ccc} \bar{u_{1}^{\prime }u_{1}^{\prime }} & \bar{u_{1}^{\prime }u_{2}^{\prime }} & \bar{u_{1}^{\prime }u_{3}^{\prime }}\\ \bar{u_{2}^{\prime }u_{1}^{\prime }} & \bar{u_{2}^{\prime }u_{2}^{\prime }} & \bar{u_{2}^{\prime }u_{3}^{\prime }}\\ \bar{u_{3}^{\prime }u_{1}^{\prime }} & \bar{u_{3}^{\prime }u_{2}^{\prime }} & \bar{u_{3}^{\prime }u_{3}^{\prime }} \end{array}\right]\;\text{where }i,j=1,2,3$$ (12)

The RANS Equation 11 present a closure problem: there are 10 unknowns (3 mean velocity components ${\bar{u}}_{1},{\bar{u}}_{2},{\bar{u}}_{3}$ , 1 mean pressure $\bar{p}$ , and 6 independent Reynolds stresses $\bar{u_{i}^{\prime }u_{j}^{\prime }}$ from the symmetric tensor) but only 4 equations (1 continuity +3 momentum). This turbulence closure problem (Orszag, 1970) requires additional modeling; here, the renormalization group (RNG) k - ε model is employed for swirl-dominated flow.

### 2.1 Numerical modeling and case setup

The Reynolds-Averaged Navier-Stokes (RANS) equations are solved numerically using the finite volume method (FVM). Pressure-velocity coupling is handled via the SIMPLE algorithm, with gradients computed using a Green-Gauss node-based scheme. A second-order upwind scheme is employed for the spatial discretization of pressure, momentum, turbulent kinetic energy, and turbulent dissipation rate.

**FIGURE 1:**
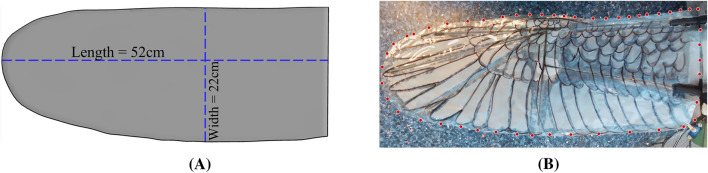
Numerical and experimental model of the bio-inspired wing. **(A)** CAD model. **(B)** Experiment model.

The computational geometry, developed in ANSYS DesignModeler, is illustrated in Figure 1. The computer-aided design (CAD) model of the bio-inspired wing (Figure 1A) is based directly on the corresponding experimental model shown in Figure 1B. The wing has a length of 52 cm, a width of 22 cm, a thickness of 0.006 cm, a planform area of 0.2 ${\text{m}}^{2}$ , and a mass of 0.016 kg.

**FIGURE 2:**
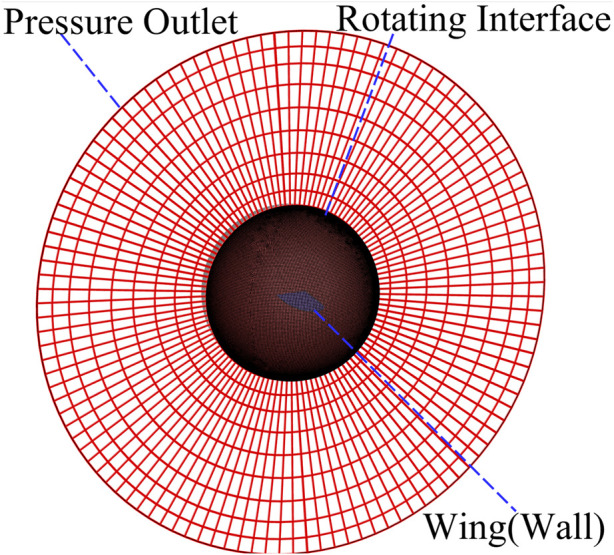
Computational mesh of the numerical model and boundary conditions.

The computational mesh is generated using ANSYS Meshing (Figure 2). The fluid domain consists of two concentric spheres. A pressure outlet boundary condition is imposed on the outer surface of the outer sphere. An interface is defined between the inner and outer spheres to accommodate the dynamic motion of the wing; this is implemented as a rotating interface using the sliding mesh technique, wherein the computational mesh remains stationary while the interface rotates. A no-slip wall boundary condition is applied to the wing surface.

**FIGURE 3:**
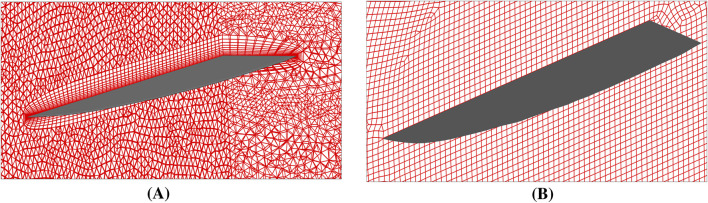
Different meshes for the computational model of the bio-inspired wing, red color shows computational mesh and grey color shows the CAD model of the wing. **(A)** 10 inflation layers mesh. **(B)** Hexahedral mesh.

To validate the numerical setup and assess mesh sensitivity, two distinct computational meshes were generated, as shown in Figure 3. The first configuration (Figure 3A) features ten inflation layers adjacent to the wall boundaries, while the second (Figure 3B) consists of a structured hexahedral mesh. Both meshes were created using ANSYS Meshing and have the same base element size.

## 3 Machine learning model

**FIGURE 4:**
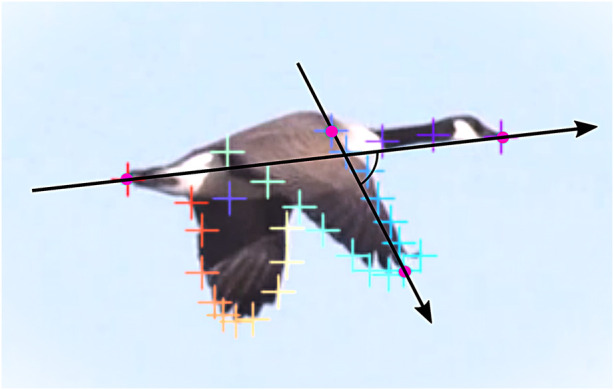
Real-time flight of a Canada goose, showing the data extracted for its flapping and twisting motion. The four pink reference points (head, wing base, tail, and wingtip) are fixed to the body and only translate with the bird. In contrast, all other tracked points are dynamic, capturing the wing’s flapping and twisting motion.

The initial dataset consisted of a video recording showing three Canada geese (*Branta canadensis*) in profile flight, sourced from iStock. To extract coordinate data from key anatomical points on the birds, DeepLabCut (DLC) Mathis et al. (2018) was employed–a deep learning-based tool designed for automated animal pose estimation. The analysis was conducted using Google Colab, a free cloud-based platform that facilitated the application of DLC to the flight digitization task. Subsequent data processing and analysis were performed in JupyterLab using Python. To ensure the reliability of the extracted flight information, keypoints were selected based on avian anatomy. Twenty frames were manually annotated within the DLC graphical interface, each containing up to 3×35 points, depending on visibility. For each bird, the set of tracked body parts comprised 35 points: the beak (1 point), neck (2 points), body (1 point), left and right wings (15 points each along the wing perimeter), and the tail tip (1 point). Using DLC, arrays of keypoint coordinates were obtained for every video frame. Individual birds were separated manually based on their X-coordinates. Clearly inaccurate or zero-valued data points were also removed manually; the remaining data were then interpolated and smoothed using Python, as illustrated in Figure 4.

### 3.1 Network architecture, training hyperparameters and data augmentation

To accommodate multiple subjects within the field of view, a bottom-up architecture based on a ResNet-50 backbone was employed, designed for simultaneous multi-instance pose estimation. The backbone was initialized with weights pre-trained on the ImageNet dataset, enabling the model to use robust feature detectors despite the limited size of the labeled dataset.

The network was instantiated as a DLCRNet with an output stride of 16, incorporating parallel prediction heads. Heatmap predictors generated probability score maps for 35 distinct body part classes, including the beak, neck segments, torso, tail, and detailed wing segmentation (8 points on the leading edge and 7 on the trailing edge for each wing). Part Affinity Fields (PAFs) encoded the spatial orientation and relationships between keypoints to support multi-animal detection, while a location refinement (LocRef) regression head predicted offset vectors to achieve sub-pixel accuracy, compensating for spatial resolution loss due to pooling operations.

Training was performed using the AdamW optimizer with a combined loss function comprising weighted binary cross-entropy for heatmap classification and weighted Huber loss for regression tasks. The model was trained for 200 epochs with a batch size of 8. A step decay learning rate schedule was applied, starting at $1\times 10^{-4}$ and reducing to $1\times 10^{-5}$ at epoch 160, and finally to $1\times 10^{-6}$ at epoch 190. To enhance generalization and account for the dynamic nature of flight, an extensive online data augmentation pipeline was employed, including random rotations within ±30° , random scaling in the range [0.5,1.25] , random crops of 448×448 pixels, and the addition of Gaussian noise to simulate varying video quality. The main parameters are presented in Table 1.

**TABLE 1:** Model architecture and training hyperparameters.

Parameter	Specification
Framework	DeepLabCut (PyTorch implementation)
Backbone architecture	ResNet-50 (pretrained on ImageNet)
Detection method	Bottom-up (DLCRNet with part affinity fields)
Input strategy	Random crops ( 448×448 pixels)
Keypoints defined	35 (beak, neck, body, tail, 15 points per wing)
Optimization algorithm	AdamW (adam with weight decay)
Loss functions	Weighted binary cross-entropy (classification)
	Weighted huber loss (regression)
Batch size	8
Training duration	200 epochs
Learning rate schedule	Step decay: $1\times 10^{-4}$ (base) $\to \;1\times 10^{-6}$ (final)
Data augmentation	Scaling (0.5–1.25), rotation (±30°) , Gaussian noise
Output stride	16

### 3.2 Flapping and twisting algorithm

**FIGURE 5:**
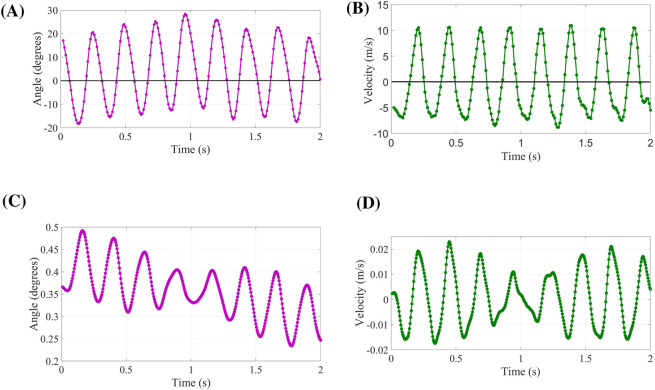
Input Signals for the wing dynamics: **(A,B)** flapping motion and **(C,D)** twisting motion over two periods.

The dynamic motion of the wing was implemented via a user-defined function (UDF) in ANSYS Fluent. This algorithm governs both the flapping stroke and the passive twisting of the wing. As previously described, a machine learning model was developed to extract the flapping and twisting kinematics from the video data. This model was integrated into the commercial CFD solver ANSYS Fluent by means of a UDF, which interprets the model predictions-specifically, the wing’s flapping angle, twisting deformation, and center-of-gravity motion, and translates them into boundary conditions for the dynamic mesh solver. The simulation was performed over two full flapping periods, during which the wing completes approximately seven oscillation cycles, as clearly demonstrated in Figure 5.

## 4 Results

**FIGURE 6:**
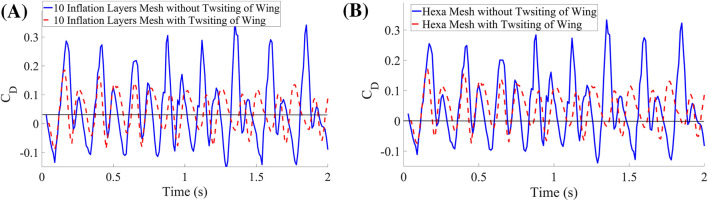
Coefficient of the drag for both cases. **(A)** 10 inflation layers mesh. **(B)** Hexahedral mesh.

This section presents the numerical results obtained from the simulations. The analysis was conducted over two complete motion periods. As shown in Figure 6, the integrated drag coefficient is positive across all cases, and the results from the four different meshes exhibit excellent agreement, indicating robust numerical convergence. The key finding is that incorporating a twisting motion during wing flapping leads to a measurable reduction in overall drag, an outcome successfully achieved in the present investigation. Furthermore, in all four mesh configurations, the time-integrated drag coefficient remains positive, confirming that net forward propulsion is sustained throughout the flapping cycle.

**FIGURE 7:**
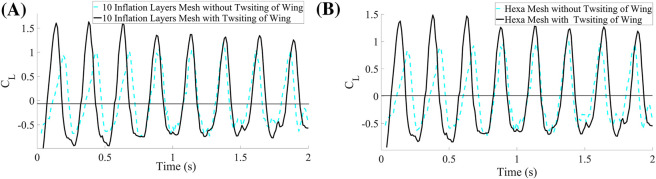
Coefficient of the lift for both Mesh Cases. **(A)** 10 inflation layers mesh. **(B)** Hexahedral mesh.

In addition, this study demonstrates that the inclusion of twisting motion substantially improves lift generation. Figure 7 clearly illustrates the increase in lift coefficient resulting from the twisting kinematics. The observed enhancement is consistent across both meshed configurations–with and without twisting–confirming that the modified motion successfully augments the aerodynamic performance of the wing.

**FIGURE 8:**
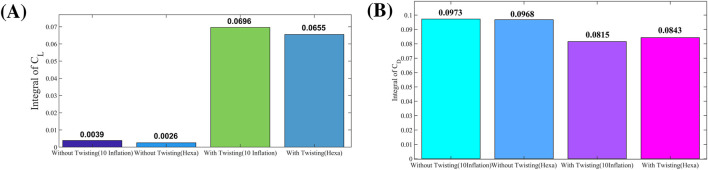
Increase in the coefficient of the lift and drag in the coefficient of the lift. **(A)** Trapezoida lintegral of the coefficient of the lift. **(B)** Trapezoidal integral of the coefficient of drag.

Initially, numerical simulations were performed on the baseline configuration, in which the wing undergoes pure flapping motion without any twisting, and the corresponding lift and drag coefficients were obtained. Following this baseline analysis, an optimized configuration incorporating wing twisting during flapping was investigated through additional simulations. A comparative analysis between the baseline and optimized cases reveals significant aerodynamic improvements attributable to wing twisting. The implementation of dynamic twisting substantially enhances lift generation while simultaneously reducing drag. Specifically, the lift coefficient demonstrates a marked increase relative to the baseline case, while the drag coefficient exhibits a noticeable reduction, as shown in Figures 8A,B. For quantitative comparison, the time-integrated aerodynamic coefficients were computed using the trapezoidal rule. The most pronounced improvement was observed in configurations incorporating dynamic wing twisting. The baseline lift coefficient of 0.003863 (corresponding to the pure flapping case without twisting) increased dramatically by 1701% and 1595%, respectively, when dynamic twisting was introduced in the two optimized cases. Similarly, the baseline drag coefficient of 0.097255 decreased by 16% and 13% in the corresponding twisted configurations. These results demonstrate that dynamic wing twisting significantly enhances aerodynamic efficiency by simultaneously increasing lift production and reducing drag.

**FIGURE 9:**
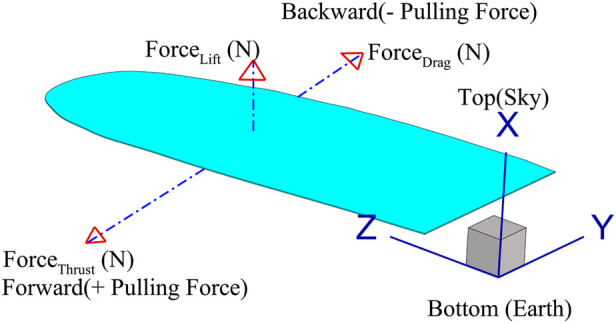
Direction of the lift, drag and thrust forces.

The lift force is also evaluated in this study and is calculated using the standard aerodynamic relation, Equation 13; the direction of the force is indicated in Figure 9.

$$Force_{\mathrm{Lift}}=C_{L}\cdot \frac{1}{2}\rho V^{2}A$$ (13)

**FIGURE 10:**
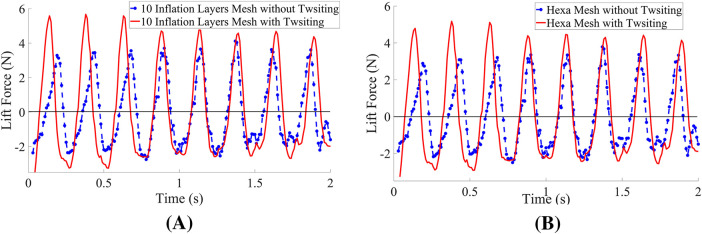
Lift Force with and without twisting motion of the wing. **(A)** 10 inflation layers mesh. **(B)** Hexahedral mesh.

Here, $C_{L}$ is the lift coefficient, ρ is the air density, V is the velocity—since no freestream velocity is present in this study, the average mean tip velocity of the flapping wing is used, and A is the wing surface area, as shown in Figure 10.

**FIGURE 11:**
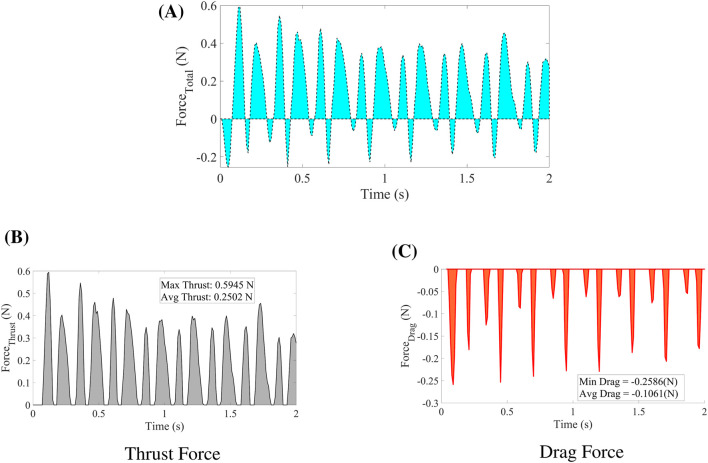
**(A)** Total force generated when the wing flapps. **(B)** Postive component of the total force (Thrust Force), that helps to move wing in the forward and **(C)** is the drag force or the negative component of the force that oppose the motion of the wing.

The results from the hexahedral mesh with twisting are used to interpret the dynamic behavior of the drag and thrust forces. The overall net force is shown in Figure 11. Positive drag, also referred to as pulling force, is presented in Figure 11B. Comparing Figures 11B,C, it is evident that the thrust force is significantly greater than the drag force. The net force is calculated using Equation 14. The distinction between thrust and drag is that drag opposes the motion of the wing (acting backward), whereas thrust propels the wing forward (acting in the direction of motion).

$$Force=C_{D}\cdot \frac{1}{2}\rho V^{2}A$$ (14)

To move the wing forward, the maximum thrust produced is 0.594 N, with an average thrust of 0.2502 N. In contrast, the maximum drag force is −0.2586 N, with an average drag of −0.1061 N.

Newton’s second law of motion provides the fundamental relationship as shown in the Equations 15–17:

F=ma (15)

where: F = thrust force vector (N), m = mass of the wing = 0.01656 kg, and a = acceleration vector (m/s^2^). The instantaneous acceleration is:

$$a\left(t\right)=\frac{F_{\text{thrust}}\left(t\right)}{m}$$ (16)

For discrete time measurements, Equation 16 becomes:

$$a_{i}=\frac{F_{i}}{m},\;i=1,2,\ldots ,n$$ (17)

where $F_{i}$ represents the thrust force at the i -th time point and the velocity is obtained by using Equations 18–20 and then by integrating acceleration over time:

$$v\left(t\right)=\int_{0}^{t}a\left(\tau \right)\;d\tau =\frac{1}{m}\int_{0}^{t}F_{\text{thrust}}\left(\tau \right)\;d\tau$$ (18)

The computation employs the trapezoidal rule for numerical integration:

$$v_{i}=v_{i-1}+\frac{a_{i-1}+a_{i}}{2}\cdot \Delta t_{i}$$ (19)

where: $\Delta t_{i}=t_{i}-t_{i-1}$ represents the time step increment, $a_{i-1}$ and $a_{i}$ denote accelerations at consecutive time points, and the initial condition is $v_{1}=0$ . Substituting Equation 17 into Equation 19 yields:

$$v_{n}=\overset{n}{\underset{i=2}{\sum }}\frac{F_{i-1}+F_{i}}{2m}\cdot \left(t_{i}-t_{i-1}\right),\;\text{with }v_{1}=0$$ (20)

**FIGURE 12:**
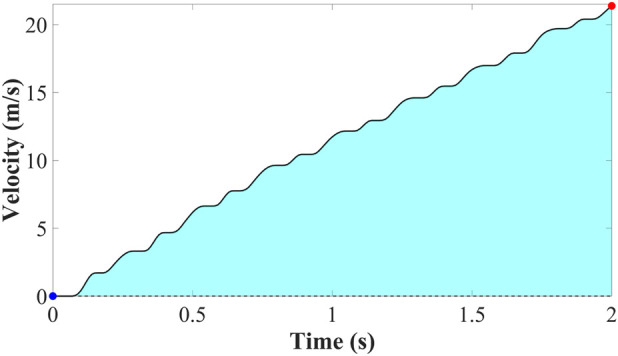
Velocity generated by thrust force.

**FIGURE 13:**
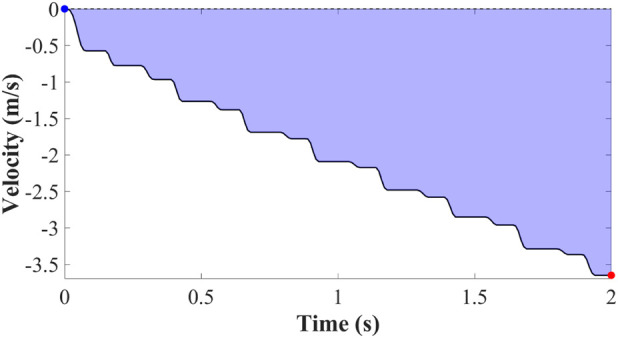
Dissipated velocity.

The velocity at each time step n is calculated from the thrust force as shown in the Figures 12, 13 history using the following relation. The key assumptions underlying this calculation are: initial velocity is zero (v(t=0)=0) ; the mass remains constant throughout the motion; thrust force is the primary contributor to acceleration; the motion is treated as one-dimensional (scalar analysis); and the time steps are sufficiently small to ensure accurate numerical integration. For one-dimensional motion,the power is defined as:

$$P\left(t\right)=F\left(t\right)\cdot v\left(t\right)$$ (21)

**FIGURE 14:**
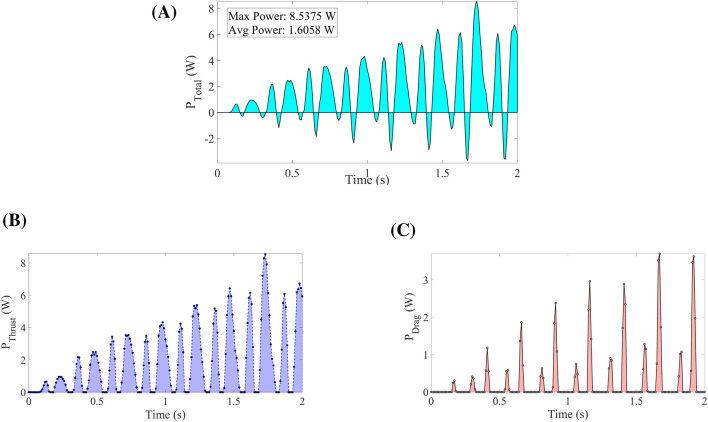
Power produced by forces: total power by Net Force **(A)**; power produced by the Thrust **(B)** and Drag **(C)** Forces.

The total aerodynamic force is decomposed into thrust (positive) and drag (negative) components Figure 14 shows, the total power genreated by the trust force and Figures 14B,C show the decomposed components of the trust force:

$$F_{\text{total}}\left(t\right)=F_{\text{thrust}}\left(t\right)+F_{\text{drag}}\left(t\right)$$ (22)

where, $F_{\text{thrust}}\left(t\right)\geq 0$ : Propulsive force in direction of motion, $F_{\text{drag}}\left(t\right)\leq 0$ : Resistive force opposing motion. Power generated by thrust force is calculated by using Equation 23:

$$P_{\text{thrust}}\left(t\right)=F_{\text{thrust}}\left(t\right)\cdot v\left(t\right)$$ (23)

This represents the rate at which thrust force does work on the object. Power dissipated by drag force is calculated by using Equation 24:

$$P_{\text{drag}}\left(t\right)=|F_{\text{drag}}\left(t\right)\cdot v\left(t\right)|$$ (24)

The absolute value ensures drag power is expressed as a positive quantity representing energy dissipation is calculated by using Equation 25.

$$P_{\text{total}}\left(t\right)=P_{\text{thrust}}\left(t\right)-P_{\text{drag}}\left(t\right)$$ (25)

**FIGURE 15:**
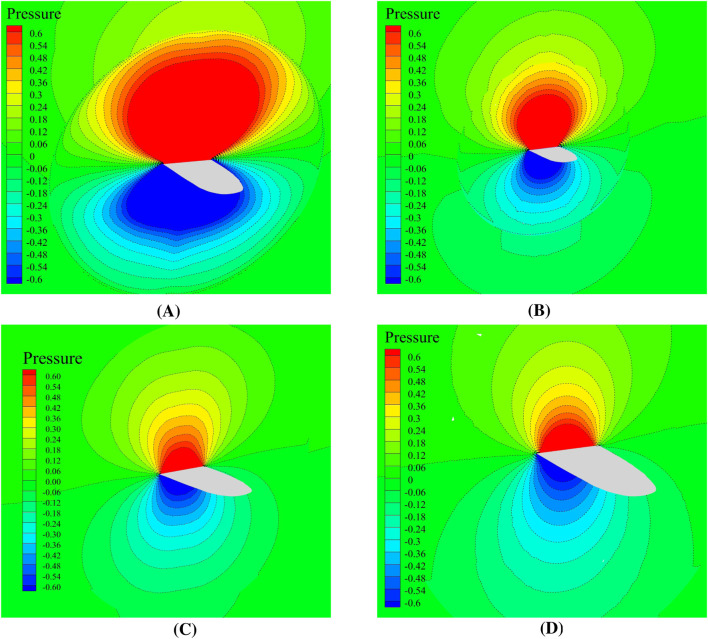
Contours of the pressure during downstroke and upstroke of 10 inflation layers mesh and Hexa mesh at T = 2s . **(A)** 10 Inflation layers Mesh without twisting. **(B)** Hexa Mesh without twisting. **(C)** 10 Inflation layers Mesh with twisting. **(D)** Hexa mesh with twisting.

The pressure contours at the end of the simulation are presented in Figure 15, with a consistent legend for direct comparison and the results in Figure 15D exhibit the greatest stability.

## 5 Conclusion

In this study, we performed comprehensive numerical simulations to investigate and optimize the aerodynamic performance of a bio-inspired flapping wing configuration. Our methodology integrated advanced computational fluid dynamics techniques with machine learning approaches, establishing a robust framework for aerodynamic analysis and design optimization.

The synergistic combination of CFD and machine learning algorithms demonstrated considerable potential for aerodynamic performance enhancement. The results exhibited strong numerical convergence and excellent agreement with established reference data and prior studies, thereby confirming the reliability and accuracy of the proposed computational framework.

Key findings indicate substantial improvements in aerodynamic efficiency metrics, including enhanced lift generation and optimized drag characteristics. The machine learning components successfully identified optimal wing configurations and kinematic parameters, while the CFD simulations provided detailed physical insights into the flow mechanisms governing performance gains.

The computational framework developed in this study can be readily extended to examine the influence of freestream velocity variations, including changes in Reynolds number, unsteady inflow conditions, and alternative wing geometries. More broadly, this work establishes a foundation for integrating data-driven methodologies with physics-based simulations in bio-inspired aerodynamic design, offering promising directions for the development of next-generation aerial vehicles and propulsion systems.

## Data Availability

The raw data supporting the conclusions of this article will be made available by the authors, without undue reservation.
